# Vocational Interests and Teaching Preferences: Who Prefers Which Teaching Topic in the Nature–Human–Society Subject?

**DOI:** 10.3390/bs13080658

**Published:** 2023-08-05

**Authors:** Angelika Pahl, Reinhard Tschiesner

**Affiliations:** Faculty of Education, Free University of Bozen-Bolzano, 39042 Brixen, Italy; angelika.pahl@unibz.it

**Keywords:** RIASEC model, interest, experience, teaching preferences, sciences, social sciences, kindergarten, primary education, student teacher

## Abstract

This study focuses on the vocational interests of trainee teachers for kindergarten and primary school, investigating whether the RIASEC-interest dimensions are related to teaching preferences in the Swiss subject of Nature–Human–Society, which is characterized by its multidisciplinarity. Interests are a source of individual differences in people and important to study since they influence intrinsic motivation, and thus, behavior, effort, and occupational decisions. The results of the conducted survey, composed of the Nature–Human–Society questionnaire and the general interest structure test (AIST-R), show, in a sample of 220 participants, that trainee teachers’ vocational interests were partly related to their previous experiences in the specific content domains of Nature–Human–Society and slightly differed by gender. The RIASEC interest dimensions of social, investigative, realistic, and partly artistic evidence significant correlations with preferences in the teaching topics of the Nature–Human–Society subject. It became clear that trainee teachers with high realistic and investigative interests and low social and artistic interests tended to prefer thing-related teaching topics, while pronounced social and artistic interests with low realistic and investigative interests were associated with teaching preferences for people-related topics in the subject of Nature–Human–Society. The dominant role of Prediger’s people- versus thing-related interest orientation could thus also be confirmed in the choice of favorite teaching topics, signaling that teachers feel comfortable with those topics that match their interest structure.

## 1. Introduction

**Figure 1:**
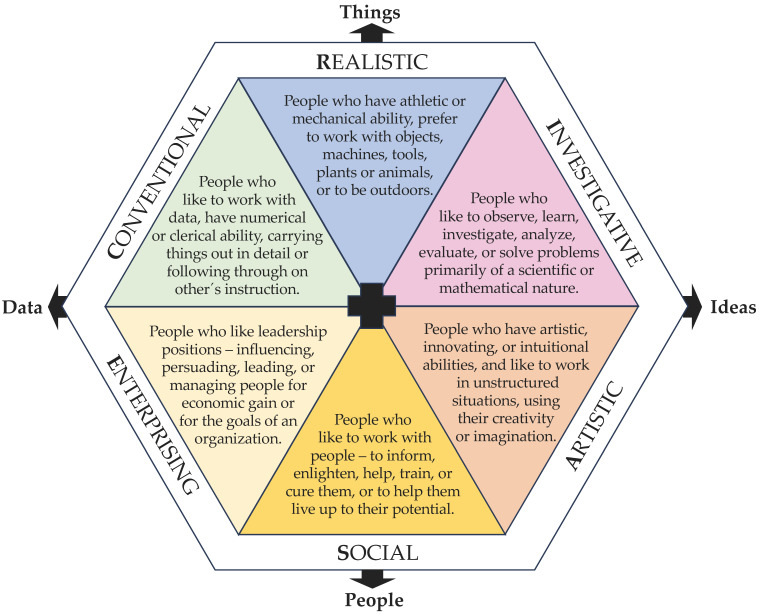
Holland´s RIASEC model with the descriptions of the six interest types [25].

Interests can be considered personality-specific characteristics that are crucial determinants of individual differences. People differ in their preferences for certain fields of knowledge or activities. Such individual interests can influence the direction, vigor, and persistence of human behaviors, as interests directly affect intrinsic motivation [1,2,3,4,5,6,7,8]. Several studies have shown that academic achievement, career choice, and job performance are influenced by interest. Strictly speaking, it is the fit between individual interests and the occupational environment that can predict career success and well-being [9,10,11,12,13]. The person–environment fit approach is prominently reflected in Holland’s theory of vocational personalities and the work environment. Holland assumed six different vocational interests that are aligned with six corresponding work environments. The six interest types are realistic (R), investigative (I), artistic (A), social (S), enterprising (E), and conventional (C), and these are collectively referred to as the RIASEC model. Each interest type is characterized by certain preferences and features, which are described in Figure 1 [14,15,16,17,18].

The RIASEC model is arranged as a hexagon (see Figure 1), suggesting that among these interest types are different relationships. Interest types that are adjacent to each other in the hexagon (e.g., social and artistic) have certain similarities or compatibilities, while opposite interest types (e.g., social and realistic) have contradictory characteristics. Each person corresponds not only to one interest type but has characteristics to differing degrees in all six of the interest dimensions, resulting in an individual personality structure [18]. 

However, usually, only the three highest expressions of the interest dimensions are used and pronounced in a three-digit Holland code, for example, SAE, which means that a person has the highest expression of interest in social, followed by artistic, and enterprising. This code of interest, SAE, is particularly common among kindergarten and primary school teachers. In Holland’s theory, the working environment of kindergarten and primary school teachers is also characterized by social, artistic, and enterprising activities and tasks. Accordingly, there would generally be a good fit between SAE personalities and this work environment. Indeed, people usually strive for such a work environment in which they can actively pursue their interests. Normally, they also have more pronounced competencies in their fields of interest, so they experience a good fit there [18,19,20,21,22,23,24]. Holland [14] mentioned that a “lack of congruence between personality (vocational interest) and environment leads to dissatisfaction, unstable career paths, and lowered performance” (p. 397). Accordingly, people tend to avoid environments that do not correspond to their interests [16].

Holland assumed, similar to Krapp [1], that interests emerge from positive experiences with activities in certain environments, which are subsequently carried out more frequently, and thus, lead to the acquisition of specific competencies [18]. According to Jantzen [26], “an experience stems from people’s interaction with their surroundings and is, as such, situated and subjective” (p. 146). Experience in terms of “something personally encountered, undergone, or lived through” [27] means a person´s evaluation of an experience in the sense of an “act or process of directly perceiving events or reality” [26] (p. 150). The subjective sensations and feelings of past events are stored in the episodic memory, in which the experiences are mentally represented. Due to the subjective quality of experiences, one and the same situation can be evaluated differently by different people [26]. Interesting situations are always accompanied by positive feelings, and individual interests can develop from situational interests [1,28,29]. Many adolescents and adults who have a pronounced interest in a particular content or activity can recall key experiences in their early childhood and school age that left a lasting impression on them [30].

Previous studies have shown that teachers’ interests can have a positive effect on teaching and pupils´ motivation. The teacher’s enthusiasm for the content of the lesson is described as contagious to the learners. It was evidenced that primary school teachers’ interest in the subject is central to the pupils’ perception of the learning contents as exciting, important, and interesting [31,32].

Swiss kindergarten and primary school teachers have a bachelor’s degree and are qualified to teach a wide range of subjects in school. The subject called “Nature–Human–Society” (“Natur-Mensch-Gesellschaft” in German) is characterized by its multidisciplinarity, as it integrates a wide variety of disciplines (e.g., physics, biology, history, geography, economics, social sciences, and religious studies) into one subject [33,34,35]. This characteristic of multi- and interdisciplinarity makes this Swiss subject different from many subject concepts in other countries, where science, history, and geography are often taught as separate subjects at the primary school level [36,37]. It also differs from other integrated subjects, such as General Studies (“Sachunterricht”) in Germany, as it incorporates more disciplines (including learning about religions, and economics) than the five content areas of “Sachunterricht” (with social and cultural, geographical, natural science, technical, and historical learning) [38]. In the Swiss subject of Nature–Human–Society, pupils engage with the world, and thus, they learn to deal with natural phenomena, different ways of life, diverse social and cultural achievements, and the spatial and economic world from different perspectives [34]. Previous studies have shown that these different disciplines are not equally popular with teachers, which, in daily teaching practice, can lead to certain topics being taught more frequently than others in this subject, and thus, some topics are often neglected [39,40,41,42,43]. For the subject of Nature–Human–Society, it was shown that for trainee teachers, the economic and physical–technical topics were among the least popular, and the biological and geographical topics were among the most popular teaching topics. Social, historical, and religious topics were in the middle field of the popularity list of teaching topics in the Nature–Human–Society subject [40,41].

Previous studies with female Swiss trainee teachers have already examined whether knowledge and ability self-concepts [43] and the Big Five personality traits [44] can predict teaching preferences in the subject of Nature–Human–Society. Vocational interests have been studied in more detail only for the physical–technical area of the Nature–Human–Society subject, showing that realistic and investigative interests are significantly associated with teaching preferences in physical–technical topics [45]. However, it is not clear whether such significant correlations with vocational interests can be found for other teaching preferences as well since vocational interest has not yet been analyzed for all content areas of the Nature–Human–Society subject. This study aims to fill this research gap because it is important to know about such possible influencing factors in teacher training to be able to take appropriate measures if necessary. Therefore, first-year students and not graduates were deliberately selected for this research. In contrast to previous studies with Swiss trainee teachers [43,44,45], this study also included male subjects in the sample in addition to female subjects, since gender differences in interests are generally well known. According to the results of many studies examining gender differences in large samples of the populations, there are two interest dimensions, namely social and realistic, which are presented quite differently between the genders; this best explains different occupational preferences, and thus, the under- or overrepresentation of men or women in certain occupational fields (e.g., women especially choose educating and health professions, while men especially choose science, technological, and engineering professions) [46,47,48,49,50,51]. Females “tend to choose occupations that are oriented towards working with people”, while males “tend to favor occupations that involve working with things” [51] (p. 210). Prediger [50] would call this distinction a people versus thing orientation. The people–thing dimension can be clearly located in the RIASEC model with the interest dimensions of realistic (thing) and social (people). In the Nature–Human–Society subject, the topics focus on both “people”, or cultural society, and “things”, or inanimate nature [34].

Interests as an individual difference domain are important to study since they are sources of intrinsic motivation, and thus, determinants of behavior, efforts, and decisions. The aim of this study was, therefore, to explore whether trainee teachers’ vocational interests (in the form of the RIASEC interest dimensions) are related to their teaching preferences in the Nature–Human–Society subject. In this way, this study pursues the aim of finding answers to the question of who prefers which teaching topics within the multidisciplinary subject of Nature–Human–Society. It was also investigated if there are gender differences in the individual interests of a trainee teachers’ group and to what extent previous experiences play a role in shaping interests.

The following research questions (RQ) are specifically addressed:

○ RQ 1: Do male and female trainee teachers for primary education differ in their expressions of experiences with the various domains of the subject of Nature–Human–Society? If so, in which areas are there significant differences?
○ RQ 2: Do male and female trainee teachers for primary education differ in their expressions of vocational interests? If so, in which areas are there significant differences?
○ RQ 3: Do male and female trainee teachers for primary education differ in their preferences of the Nature–Human–Society teaching topics? If so, in which areas are there significant differences?
○ RQ 4: Is there a (positive) correlation between trainee teachers’ experiences of the Nature–Human–Society domains and their expressions in vocational interests? If so, in which areas are there very significant relationships?
○ RQ 5: Is there a (positive) correlation between the popularity of the Nature–Human–Society teaching topics and the expression of vocational interests among trainee teachers? If so, in which areas are there very significant relationships?

## 2. Materials and Methods

### 2.1. Sample

The study intended to examine almost the entire cohort of first-semester students of the primary education program at the University of Teacher Education Bern (Switzerland). In the academic year in which the study was conducted, the overall cohort was composed of 83% women and 17% men. No exclusion criteria were defined, to obtain a sample as representative of reality as possible, to ensure that the study had high external validity.

A total of 220 students participated in the entire survey. This represents 71.2% of the first-year cohort composed of 309 students. Of the remaining 28.8% of the cohort, 36 students (11.7%) did not take part in the survey at all, while 53 students (17.2%) began the survey but did not complete it in full, so they could not be considered for the sample.

The sample consisted of 84.1% women and 15.9% men and had an average age of 23.26 years (*SD* = 6.3; *Min* = 18; *Max* = 50), with 70.0% of the participants being between 18 and 22 years old. A total of 59.6% of the participants had a high school diploma (of which 10% already had a university degree), while the others had a vocational diploma, a specialized diploma, or basic vocational training and had passed an admissions exam to enroll in the study program. A total of 44.1% of the sample opted for a study focus on cycle 1 (teaching kindergarten to grade 2) and 55.9% for a study focus on cycle 2 (teaching grades 3 to 6).

### 2.2. Data Collection and Instruments

The survey was conducted online using the survey software SWITCH, which is provided by the University of Teacher Education Bern. As part of the trainee teacher course for Nature–Human–Society, all first-semester students were invited via email to participate in the survey during the first week of the academic year and had one week to complete the survey. Participation was voluntary and anonymous. The online survey lasted approximately 20 min and consisted of three questionnaires, which are described in further detail below.

#### 2.2.1. Q-Sort: Favorite Content to Teach within the Subject of Nature–Human–Society

From a list of twelve teaching topics on the subject of Nature–Human–Society, the students were asked to choose three topics that they would most like to teach and to indicate their rankings as 1, 2, or 3. The same list was also used to ask students which three teaching topics they disliked the most, disliked the second most, and disliked the third most. The other, not-selected teaching topics formed the middle field, i.e., these were the teaching topics that they neither particularly liked nor particularly disliked [52].

#### 2.2.2. Nature–Human–Society Questionnaire of Experience (NMG Questionnaire)

The NMG Questionnaire is a standardized self-report instrument for Swiss kindergarten and primary school trainee teachers and in-service teachers. It focuses on the seven content areas of the Nature–Human–Society subject, namely social–ethical, cultural–religious, historical–political, geographical, economic, physical–technical, and biological.

In this study, only the experience scale of the NMG Questionnaire was considered. The experience subscale recorded the students’ school experiences on the content areas in terms of whether they were positive, whether they had also dealt with them and become familiarized with them outside of school, and whether they associated these content areas with positive memories. The participants were asked to answer the same three questions on the experience scale for each of the seven NMG content areas, rating a total of 21 items on a 5-point Likert scale (1 = strongly disagree; 5 = agree strongly). The Cronbach´s alphas of the experience subscales for all the content areas of the Nature–Human –Society subject were indicated by the questionnaire evaluators ranging between 0.68 and 0.77 [53].

#### 2.2.3. General Interest Structure Test (AIST-R)

The AIST-R (Allgemeiner-Interessen-Struktur-Test Revision in language name) is a self-report questionnaire based on the RIASEC model. The six RIASEC-interest dimensions were expressed by the participants by rating 10 items each on a 5-point Likert scale (1 = I am not interested in that at all; 5 = I am very interested in that). The RIASEC-interest dimensions are realistic, investigative, artistic, social, enterprising, and conventional, and thus, capture Holland’s six personality types. The AIST-R was created by Bergman and Eder in German and is considered a validated and reliable self-assessment instrument. The Cronbach´s alphas of all the AIST-R scales were cited by the test developers as ranging between 0.82 and 0.87. The manual contains standards (*N* = 2496) and an occupational register with assigned RIASEC codes that characterize the respective work environment [18].

### 2.3. Data Analyses

The raw data of the online questionnaire were exported to an SPSS file. Before beginning the analyses in SPSS, the raw data were pre-screened to detect any error outliers and random tick marks. Where indicated by the questionnaires, scales were formed from related items, and the respective Cronbach’s alphas were calculated. After that, descriptive statistical analyses were performed focusing on the sample composition, the questionnaire results on experience in the Nature–Human–Society domains and vocational interests, and the ranking of teaching topics according to like and dislike. The sample was also divided into male and female subsamples to identify possible gender differences (RQ 1, 2, and 3). In order to exclude further inhomogeneities in the sample, possible differences between participants with a major in cycles 1 and 2, as well as between participants with and without a high school diploma, were examined in a preliminary analysis. However, no significant differences were found, so these demographic variables were not considered further in the analysis of this study. For the descriptive comparability of the six vocational interest dimensions, the raw data were transformed into standard values according to the specifications in the manual. In addition, the scales for the experiences in the seven content domains of Nature–Human–Society were z-transformed in order to be able to classify which domain-related experiences were more or less pronounced in comparison to the overall experience in Nature–Human–Society.

In general, non-parametric tests were performed for variables with a rank-scaled level, but also with an interval scale level to prevent the results from being affected by a violation of the distribution. Thus, for the research questions regarding gender differences (RQ 1, 2, and 3), significance tests for two independent samples were calculated using the Mann–Whitney *U* test. To interpret the effect size, Cohen’s *d* was calculated for any gender difference between the variables with an interval level. To answer the research questions regarding possible correlations of the variables, namely experiences and vocational interests (RQ 4) and vocational interests and teaching topic popularity (RQ 5), the Spearman’s rho values were calculated for correlation analyses [54,55,56].

## 3. Results

### 3.1. Trainee Teachers’ Experiences about Nature–Human–Society Domains (RQ 1)

Cronbach´s alphas for the experience scales, composed of three items for each Nature–Human–Society domain, ranged from 0.794 to 0.869. In Table 1, the results of each scale are presented, considering the whole group as well as the female and male subgroups.

A Mann–Whitney *U* Test was carried out to determine if there were gender differences in the seven Nature–Human–Society domains of the experiences scales. There was a statistically significant difference in the social–ethical experience between the groups (*U* = 2164.500, *Z* = −3.134, *p* < 0.005, *d* = 0.581), as well as in the physical–technical experience (*U* = 2247.000, *Z* = −2.885, *p* < 0.005, *d* = −0.537) and the economic experience (*U* = 2496.500, *Z* = −2.165, *p* < 0.05, *d* = −0.308). Compared to males, females had significantly higher scores in social–ethical experience, while females’ physical–technical and economic experience scores were significantly lower than those of males. The results of biological experience (*U* = 2645.000, *Z* = −1.739, *p* = 0.082, *d* = 0.284), cultural–religious experience (*U* = 2899.500, *Z* = −0.986, *p* = 0.324), historical–political experience (*U* = 2978.000, *Z* = −0.756, *p* = 0.450), and geographical experience (*U* = 2810.500, *Z* = −1.247, *p* = 0.212) did not indicate significant differences between females and males.

**Figure 2:**
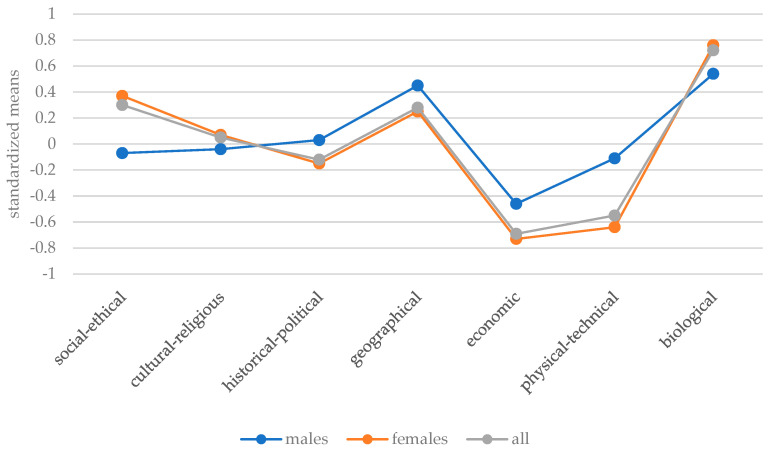
Standardized means of experience of all, female, and male participants.

To make the experience scores of different domains comparable, standard values were calculated and are shown separately in Figure 2 for all participants, females, and males. Here it becomes clear once again that the females’ experiences in the areas of biology, geography, and sociology–ethics were very positive, while their experiences in the areas of economics and physics–technology were clearly negative. The experiences of men in the various Nature–Human–Society domains did not differ as much overall from those of women, but they also tended to show more negative experiences in the domains of economics and physics–technology and positive experiences in the domains of biology and geography.

### 3.2. Trainee Teachers´ RIASEC Interest Dimensions (RQ 2)

The RIASEC subscales produced Cronbach´s alphas between 0.785 and 0.858. Table 2 presents the results of the six interest scales separately for all participants, females, and males.

Mann–Whitney *U* tests were performed to evaluate whether the RIASEC interest dimensions differed between females and males. The results indicate that females had significantly higher social and artistic interests than males (*U*_social_ = 1288.500, *Z* = −5.653, *p* < 0.001, *d* = 1.167 and *U*_artistic_ = 1625.500, *Z* = −4.671, *p* < 0.001, *d* = 0.948). Additionally, there were significant gender differences in realistic and conventional interests (*U*_realistic_ = 2258.000, *Z* = −2.840, *p* < 0.01, *d* = −0.528 and *U*_conventional_ = 2359.500, *Z* = −2.546, *p* < 0.05, *d* = 0.445). Males had significantly higher scores in realistic and lower scores in conventional interest than females. Non-significant differences between both groups were evidenced for investigative and enterprising interests (*U*_investigative_ = 2978.000, *Z* = −0.752, *p* = 0.452 and *U*_enterprising_ = 2876.500, *Z* = −1.047, *p* = 0.295).

**Figure 3:**
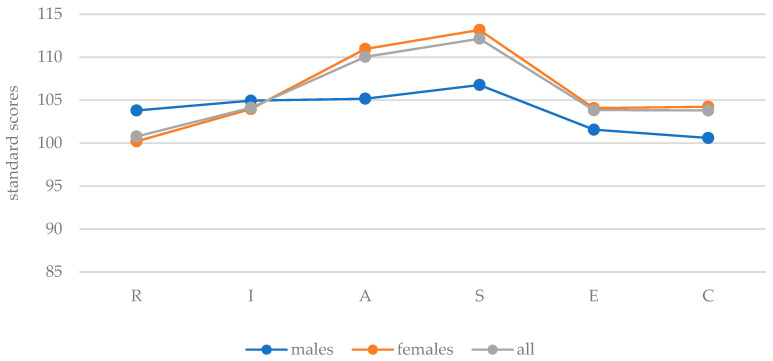
RIASEC standard scores of the means of all, female, and male participants.

The results of the individual RIASEC scales were converted into the general norm scores and are presented in Figure 3. The female participants showed the highest scores in social, artistic, and enterprising interests (SAE profile). The males also had social and artistic interests, but then investigative interest came third (SAI profile). Overall, it is noticeable that the differences between the RIASEC interest dimensions were smaller among males than among females. For the female participants, the social and artistic interests were clearly above the overall norm. The lowest levels of interest were conventional interest for men and realistic interest for women.

### 3.3. Trainee Teacher’s Selection of Favored Teaching Topics (RQ 3)

In Table 3, the teaching topics are ranked according to popularity. On the left of the table, the rankings are given according to the frequency with which the teaching topics were mentioned as the first, second, or third favored teaching topic, while on the right, the rankings are given according to the frequency with which the teaching topics were mentioned as the first, second, or third disliked topic to teach. The rankings are shown for the total sample as well as for women and men separately.

Mann–Whitney *U* tests were used to evaluate whether the selection of the popularity of teaching topics differed between females and males. Two statistically significant group differences were evident: Compared to males, females expressed less popularity for the teaching topic “Technology, electricity, and inventions” (*U* = 1664.000, *Z* = −4.704, *p* < 0.001) and more popularity for the teaching topic “Thinking about oneself” (*U* = 2533.000, *Z* = −2.191, *p* < 0.05). There were no significant group differences in the popularity ranking of other teaching topics: “Being with others” (*U* = 2968.500, *Z* = −0.872, *p* = 0.383), “Religions and traditions” (*U* = 3091.000, *Z* = −0.450, *p* = 0.652), “How products were produced” (*U* = 2838.500, *Z* = −1.328, *p* = 0.184), “Life in earlier generations” (*U* = 3160.000, *Z* = −0.235, *p* = 0.814), “My hometown” (*U* = 3010.500, *Z* = −0.716, *p* = 0.474), “Earth, and how people live in other places” (*U* = 3133.000, *Z* = −0.325, *p* = 0.746), “Animals, woods, fields, ponds, and flowers” (*U* = 2951.000, *Z* = −0.887, *p* = 0.375), “Sun, moon, stars, and universe” *U* = 2898.000, *Z* = −1.091, *p* = 0.275), “Water, air, weather, and stones” (*U* = 3189.000, *Z* = −0.166, *p* = 0.868), and “Substances and their properties” (*U* = 2919.500, *Z* = −0.958, *p* = 0.338).

**Figure 4:**
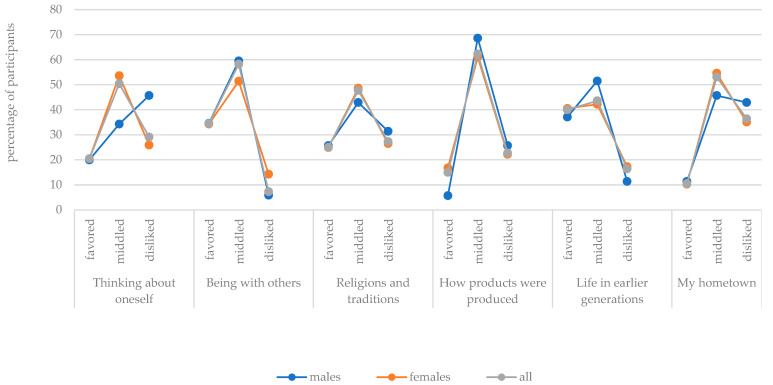
Percentages of participants—separate for males (*n* = 35), females, (*n* = 185), and all (*n* = 220)—who selected the various teaching topics as favored, middled, and disliked (part I).

**Figure 5:**
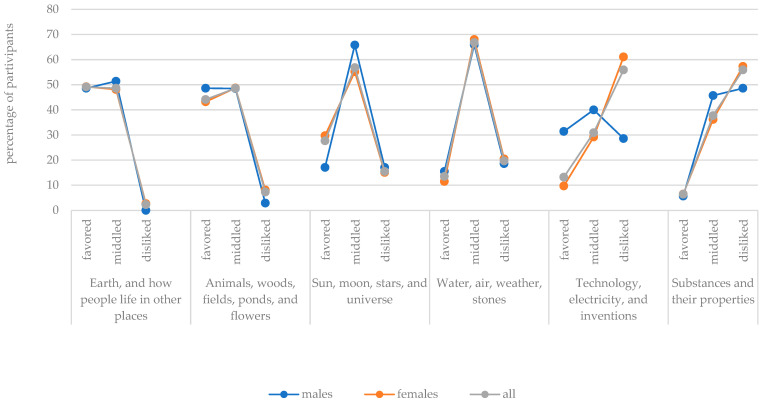
Percentages of participants—separate for males (*n* = 35), females, (*n* = 185), and all (*n* = 220)—who selected the various teaching topics as favored, middled, and disliked (part II).

The graphs in Figure 4 and Figure 5 illustrate the percentages of trainee teachers who favored, middled, or disliked the different teaching topics. If the whole sample is considered, the graphs of the topic “Earth, and how people live in other places” and the topic “Animals, woods, fields, ponds, and flowers”, evidence a right-skewed form. This indicates that these topics were very popular. On the other hand, the graphs that describe the popularity of the topics “Substances and their properties”, “My hometown”, and “Technology, electricity, and inventions” evidence a left-skewed form, which means that they were less popular.

If the sample is divided by gender, the popularity corresponded in all cases except for two specific topics. The graphs describing the “Technology, electricity and inventions” topic illustrated that only half as many male subjects as female subjects disliked this topic and that, in contrast to females, there were also more male participants who favored this teaching topic. Furthermore, the graph of “Thinking about oneself” indicates that this topic was nearly equally popular and unpopular among female trainee teachers, while the graph of the male subgroup clearly points in the direction of unpopularity.

Overall, it must be noted that the sample graph of all participants is, in all cases, more similar to the female graph than to the male graph because the female subjects are represented in the whole sample with a larger proportion. In addition, it is noticeable that most graphs show the highest percentages in the middle range of the popularity of the teaching topic.

### 3.4. Correlation with RIASEC Interest Dimensions (RQ 4 and RQ 5)

Spearman’s rank correlation was computed to assess the relationship between the experience variables in the seven Nature–Human–Society domains and the RIASEC interest variables. As shown in Table 4, the strongest positive correlations were found between physical–technical experience and investigative interest and between physical–technical experience and realistic interest. Social–ethical experience and cultural–religious experience were correlated highly respectively very significantly and positively with social interest and artistic interest. Additionally, there were highly significant and positive correlations between historical–political experience and artistic interest, and between economic experience and conventional interest. Biological experience correlated positively and highly significantly with investigative interest, as well as very significantly with social interest, and realistic interest. The results shown in Table 4 also evidence further significant correlations as well as non-significant associations.

Spearman’s rank correlations were also calculated between the RIASEC interest variables and the teaching topics’ popularity variables (see Table 5). The popularity scale of the teaching topic “Technology, electricity, and inventions” was found to be highly respectively very significant and positively correlated with realistic and investigative interests, while it was highly respectively very significant but negatively correlated with artistic and social interests. Moreover, the popularity of the teaching topic “Substances and their properties” correlated highly significantly and positively with investigative interests, while very significant and negatively with social interest. There was a positive, highly significant correlation between the popularity scale of the teaching topic “Being with others” and social interest. A very significant but negative correlation was found between realistic interest and the teaching topics “Thinking about oneself” and “Life in earlier generations”. Further significant and non-significant associations between variables are shown in Table 5.

## 4. Discussion

The outcomes of this research provide insight into trainee teachers’ vocational interests and demonstrate their relationships with prior experiences with the Nature–Human–Society content areas, as well as with current teaching preferences in the Nature–Human–Society subject.

Starting with the results of the experiences (RQ 1), it can be stated that male and female trainee teachers for primary education had quite similar experiences with the content areas of the Nature–Human–Society subject, except for social science, economics, and physics–technology, where the former experiences were more positively pronounced in women, and the latter two in men. The effect sizes of these differences could be interpreted as low to medium. Generally, it was noticeable that the experiences with biology and geography were generally positive and above average, while those with economics and physics–technology were below average compared to the overall experience scale of the Nature-Human-Society subject. The experience outcomes of the female subsample were essentially consistent with those of another sample of Swiss female trainee teachers [45], although, in the latter, the experience scores in geography were slightly lower.

Exploring the question of whether male and female trainee teachers’ vocational interests differed (RQ 2), the general answer is yes, except for the two interest dimensions of investigative and enterprising, where no significant differences were found. The social interest was highest for both genders, indicating a good fit with the teaching profession. In previous studies, social interest was also described as the best predictor for entering the teaching profession [20,23]. In the comparison between male and female trainee teachers in the sample of our study, however, social interest was once again significantly higher among women. In addition, there was a demonstrated gender difference with higher scores in females for the artistic interest dimension, which, according to Holland is also considered a crucial interest dimension for the profession of kindergarten and primary school teachers. In the male subsample, however, artistic interest was still well above the mean of the overall standard [18] and among the three highest expressions of interest. In the case of female teachers, the third highest interest dimension was enterprising, and thus, the typical SAE profile for kindergarten and primary school teachers was optimally fulfilled according to the literature [18,23]. In contrast, if we look at the male subsample, it was the investigative interest that emerged as the third highest interest dimension. The SAI profile, as shown by the male subsample, is often found among people working as secondary school teachers, psychologists, social pedagogists, psychotherapists, and nurses [18]. Thus, even the male subsample still showed a clearer people orientation than a thing orientation, as named by Prediger, although, in general, the male gender is more likely to be found in occupations that are thing-oriented [46,47,48,49,50,51]. With the interest orientation that the male participants showed, they are placed well in occupations with people orientation. Nevertheless, it is noticeable in the results that the realistic interest, which is a characteristic of thing orientation, was higher in the male than in the female subsample. The effect sizes of the gender differences could be classified as high for the social and artistic interests, whereas the effect sizes regarding the gender difference in the realistic interest could be interpreted as medium, and those for the conventional interest as low. The higher the effect size, the higher the probability that the differences are not only numerical but also phenomenological. Phenomenologically, differences could, therefore, be particularly apparent for social and artistic interests.

Regarding the question of whether gender differences were also evident for teaching preferences in the Nature–Human–Society subject (RQ 3), it can be said that more similarities than differences were found. A gender-specific effect could only be demonstrated for the teaching topics of “Thinking about oneself” and “Technology, electricity, and inventions”, where the former topic can be classified as people-oriented and the latter as thing-oriented. Consequently, it is not surprising that the former was preferred significantly more often by women and the latter by men, especially since it was also shown that the men subsample had a significantly higher realistic (thing-orientated) and a significantly lower social (people-oriented) interest than the female subsample. For the other topics, there was reasonably clear agreement in terms of likes and dislikes, in which there is a tendency for people-related topics to be more popular than thing-related topics. Thus, thing-related topics or topics of inanimate nature (such as “How products are produced”, “Water, air, weather, and stones”, “Technology, electricity, and inventions”, and “Substances and their properties”) were ranked at the bottom of the popularity scale. However, at the top of the popularity list, there were also not clearly people-related topics, but topics that refer to animate nature in general, such as animals and plants, or mixed topics, such as “Earth, and how people live in other places”, which is attributable to both physical and human geography. Immediately after those favored topics in the ranking, however, come people-related teaching topics such as “Life in earlier generations” and “Being with others”. The teaching topic favorite list of this sample is broadly consistent with the teaching topic favorite list of female teacher candidates from a previous study [41].

In the following, the correlations between the participants’ vocational interests and previous experiences (RQ 4) and their current teaching preferences (RQ 5) are discussed in more detail. To this end, it must first be noted that here, in the analyses of the correlations, only those significant scores that turned out to be very or highly significant (*p* < 0.01 or *p* < 0.001) are interpreted.

We start with the connections between experience and interest (RQ 4), which, according to the literature, should generally be given [13]. The strongest relationships between previous experiences and vocational interests found in this study were all positive, meaning that the more positive experience in a content area of the NMG subject, the more pronounced the corresponding vocational interest was. Better and more experience in a content area may also be associated with more distinctive skills, as Holland postulated in his theory [16,18]. The relationship between experience in the physical–technical area and realistic interest can be explained well if it is known that realistic interest is characterized by people who like to work with materials and tools and engage in hands-on activities with tangible results. The prototypical realistic personality type often has highly evolved mechanical, technical, electrical, and agricultural skills. Furthermore, realistic types show a tendency to prefer working outside and with animals and also have good skills in working with plants and animals [14,15,16,17,18]. These characteristics also make it easy to understand the connection between positive experiences in biology and realistic interest. The experiences in the biological as well as physical–technical domains are also correlated highly significantly with investigative interest. This dimension of interest, investigative, also corresponds theoretically well with such positive experiences in the natural–technical sciences since it is characterized by competencies in and preferences for solving problems in the field of science and engineering. Moreover, the two vocational interests of realistic and investigative are also arranged side by side in Holland’s RIASEAC model, which signals a high degree of coherence between these interest dimensions [14,15,16,17,18]. When we look further at the results of this study, it becomes clear that even more strong relationships between experiences and vocational interests were revealed. Positive and frequent experiences in the social–ethical, cultural–religious, and historical–political fields correlated positively with artistic interest. Since artistic interest is characterized by preferences in the self-expression of ideas and creative activities in different cultural arts, these strong relationships are not unplausible. “Cultural arts … are tools that help develop the mind and body, refine feelings, and thoughts and reflect and represent our customs and values as a society” [57] (n. p.). Artistic interest types like to be expressive and deal with ideas as well as people. Ideas and people typify the two adjacent interest dimensions of investigative and social interests, and therefore, artistic interest exhibits a high degree of coherence with them [16,18,48,50]. Due to their coherent fit in the interest dimensions, it is also understandable that experiences in the social–ethical and cultural–religious fields are also positively correlated with social interest. The social interest dimension can be theoretically well related to the experiences in the field described above since social interest types are interested in social and ethical issues and have special skills in interpersonal relationships and social interaction. In addition, social interest types are concerned with the welfare of others and feel comfortable in an environment where they can engage with others to make a positive impact [14,15,16,17,18]. Continuing with further results of the correlation analyses, a highly significant correlation between experience in the field of economics and conventional interests must still be noted. Theoretically, this relationship can be explained well, since conventional interest types are interested in processing economic data and have skills in accounting, management, and business. They prefer structured business activities and value success in business, and therefore, experience in this field is certainly beneficial [9]. Finally, the fact that there were no significant relationships between vocational interests and experience in the geographical area may be because experiences in this area are not so specific and pertinent for the dimensions of vocational interest.

In the last section of the discussion, we will clarify the question of whether very significant relationships can also be found between vocational interests and teaching preferences in the Nature–Human–Society subject (RQ 5). The vocational interest dimensions of social, investigative, realistic, and artistic evidenced strong correlations with some teaching topic preferences in the subject of Nature–Human–Society, while the interest dimensions of enterprising and conventional did not. Prediger described enterprising and conventional interests as typically relevant for data-related work tasks [48,50], and they do not seem to play a role in the selection of favored teaching topics. The people–thing dimension, which Prediger located within Holland’s RIASEC model as an axis between realistic and social interest [48,49,50], on the other hand, appeared to be sometimes significant in assigning teaching topics by popularity. The analyses showed that very or highly significant correlations with people-related topics had either a negative correlation with realistic interest or a positive correlation with social interest. It can be recorded in detail that the teaching topics of “Thinking about oneself” and “Life in earlier generations” were favored by trainee teachers who showed a comparatively low level of realistic interest, while the teaching topic of “Being with others” was favored by trainee teachers who showed a comparatively high level of social interest. Vice versa, it turned out that the thing-orientated teaching topics of “Technology, electricity, and inventions” and “Substances and their properties” were favored by trainee teachers with high levels of investigative interest and low levels of social interest. For the former teaching topic, namely “Technology, electricity and inventions”, two further dimensions of interest were decisive: The teaching topic was favored by trainee teachers who also had high levels of realistic and low levels of artistic interest. Thus, in summary, it became clear that trainee teachers with high realistic and investigative interests and low social and artistic interests tended to prefer thing-related teaching topics, while pronounced social and artistic interests with low realistic and investigative interests were associated with teaching preferences for people-related topics in the Nature–Human–Society subject. According to Holland’s theory, the patterns of interest that we found in highly or very significant correlations with the mentioned teaching topics are coherent in themselves. Realistic and social interests are arranged opposite each other in the RIASEC model and are understood to be opposites, whereas realistic and investigative as well as social and artistic interests are placed right next to each other, and thus, have high coherence [14,15,16,17,18]. The dominant role of Prediger´s people-versus-thing orientation could thus also partly be confirmed in the choice of favorite teaching topics, signaling that teachers feel comfortable with those topics that match their vocational interests. However, not all preferences of teaching topics in the Nature–Human–Society subject were related to vocational interests. Preferences can, therefore, also be based on other reasons or beliefs, e.g., because the trainee teachers think that these teaching topics are particularly exciting or feasible for children, because they are particularly meaningful for the children, or because of their perceived low or high capability in teaching those topics. Further work is certainly required to disentangle the complex influences on teaching preferences. The use of qualitative methods in addition to quantitative ones could be especially promising for analyzing trainee teachers’ subjective arguments for preferring or rejecting certain teaching topics in detail. It would also be interesting to conduct longitudinal studies to examine whether and how teacher training influences teaching preferences in the Nature–Human–Society subject.

## 5. Conclusions

It is important to note that our results are based on the voluntary participants of one trainee teacher cohort. Therefore, the generalizability of the conclusions of this study is limited. In particular, it would be desirable to enlarge the male subsample with additional cohorts, since gender differences turned out to be significant in this study, and men were represented with a rather small proportion. This means that the statements about the male group in this study are extremely sample-dependent and must be interpreted carefully. However, the underrepresentation of men in teacher training for kindergarten and primary school is typical, and the characteristic gender ratio of these trainee-teacher cohorts is well reflected in the sample, so the sample can be considered representative [58].

All in all, it can be concluded from the study that the importance of vocational interests in teacher education should be recognized and that positive experiences should be provided in teacher training that can foster trainee teachers’ interests in teaching all topics of the Nature–Human–Society subject. The study has in fact made clear that prospective teachers generally prefer to teach people-oriented topics than thing-oriented topics. This decision-making behavior carries the risk that thing-oriented topics are not addressed as frequently in everyday teaching of Nature–Human–Society subjects and, consequently, pupils cannot gain as many learning experiences in these areas as in people-oriented areas.

Nevertheless, it is encouraging that the social interest of trainee teachers, independent of gender, has been identified as the highest. Thus, it is an inner need of trainee teachers to work with other people, in this case, specifically to teach children and help them learn and grow. In teacher education, trainee teachers take on a change in role, away from the role of the student to the role of the teacher. In this way, teaching topics are also grasped from a different perspective, not only from a subject-specific perspective but also from a subject-didactic perspective [35]. This also means that the teaching of certain topics represents a different sphere of interest than the topic itself, which they have experienced in the past [59]. Thus, teacher training could also try to give trainee teachers access to rather unpopular topics by showing them how to teach them. It is well established that children in kindergarten and primary school are very interested in topics of inanimate nature, and they want to explore and investigate the environment and also want to learn more about thing-related topics [60,61]. Certainly, trainee teachers with high social interest want to support children in this learning and exploring. In teacher training, we can show how they can do this. Maybe their previous beliefs toward teaching certain topics do not fit with the current didactic ideas for kindergarten and primary school, and child-centered approaches to these topics can also inspire them.

Since people-oriented topics are known to be more popular with trainee teachers than thing-oriented topics, a connection can generally be made between these areas by embedding thing-oriented topics in personally and socially significant contexts. By showing trainee teachers the fields of application of thing-oriented topics and thus the benefits for people, the learning and teaching of such topics can become more interesting and meaningful [62,63,64].

Ultimately, it is important that teacher education awakens a situational interest in trainee teachers for teaching a wide variety of content, especially for topics previously classified as unpopular. Teacher educators, therefore, need to create appealing and practice-relevant learning situations that evoke positive responses and a sense of competence in trainee teachers [1,7,8,28]. Such positive experiences can change individual interests in teaching formerly unpopular topics in the long term. The curriculum of teacher education and the teacher educators thus play a crucial role because the former creates the opportunity for competence building and interest development and the latter concretely implements it.

Overall, it can be stated that prospective teachers should develop a professional understanding of their future professional role during their training and understand that teaching is not about their personal interests, but that teaching and learning in kindergarten and primary school must be oriented towards the interests of children in the different educational areas that the curricula specify.

## Figures and Tables

**Table 1:** Cronbach´s alphas, means, and standard deviations of experiences in the different Nature–Human–Society domains (the experience Likert scales ranged between 1 = low and 5 = high).

Experience	α (*n* = 220)	*M(SD)*_all_ (*n* = 220)	*M(SD)*_Females_ (*n* = 185)	*M(SD)*_Males_ (*n* = 35)
Social–ethical Cultural–religious Historical–political Geographical Economic Physical–technical Biological	0.841 0.807 0.794 0.847 0.836 0.869 0.828	3.66 (0.87) 3.41 (0.93) 3.23 (0.96) 3.63 (0.88) 2.65 (0.89) 2.79 (1.00) 4.09 (0.79)	3.73 (0.89) 3.42 (0.95) 3.20 (0.99) 3.60 (0.89) 2.61 (0.93) 2.70 (0.98) 4.12 (0.80)	3.28 (0.66) 3.31 (0.84) 3.38 (0.78) 3.81 (0.82) 2.89 (0.65) 3.24 (1.03) 3.90 (0.75)

**Table 2:** Cronbach´s alphas, means, and standard deviations of the RIASEC interest dimensions.

RIASEC	α (*n* = 220)	*M(SD)*_all_ (*n* = 220)	*M(SD)*_Females_ (*n* = 185)	*M(SD)*_Males_ (*n* = 35)
Realistic (R) Investigative (I) Artistic (A) Social (S) Enterprising (E) Conventional (C)	0.838 0.830 0.785 0.858 0.806 0.830	27.78 (6.93) 32.12 (6.67) 37.04 (6.47) 40.16 (6.08) 32.84 (6.30) 27.82 (6.47)	27.21 (6.85) 31.97 (6.80) 37.96 (6.14) 41.18 (5.68) 33.08 (6.37) 28.24 (6.64)	30.80 (6.73) 32.94 (5.90) 32.17 (6.08) 34.77 (5.30) 31.57 (5.84) 25.60 (5.21)

**Table 3:** Ranking positions of Nature-Human-Society teaching topics for favored and disliked selection; separately listed for all participants (*n* = 220), females (*n* = 185), and males (*n* = 35).

Ranking: Favored All/Females/Males	Teaching Topics	Ranking: Disliked All/Females/Males
1/1/1 2/2/2 3/3/3 4/4/4 5/5/8 6/6/6 7/7/7 8/8/11 9/9/9 10/11/5 11/10/10 12/12/12	Earth, and how people live in other places Animals, woods, fields, ponds, and flowers Life in earlier generations Being with others Sun, moon, stars, and universe Religions and traditions Thinking about oneself How products were produced Water, air, weather, and stones Technology, electricity, and inventions My hometown Substances and their properties	12/12/12 11/10/11 8/7/9 10/9/10 9/8/7 5/11/4 4/4/2 6/5/5 7/6/6 2/1/8 3/3/3 1/2/1

**Table 4:** Correlations between variables of RIASEC and experiences in the NMG domains, *r*(118).

Experiences in NMG Domains	R	I	A	S	E	C
Social–ethical	–0.13 *	–0.03	0.35 ***	0.46 ***	0.17 *	0.01
Cultural–religious	–0.06	–0.04	0.24 ***	0.20 **	0.12	0.02
Historical–political	–0.15 *	–0.04	0.25 ***	0.02	0.07	–0.06
Geographical	0.07	–0.04	0.00	0.04	–0.03	0.04
Economic	0.15 *	0.15 *	0.00	–0.14 *	0.17	0.24 ***
Physical–technical	0.51 ***	0.50 ***	–0.16 *	0.11	–0.08	0.15 *
Biological	0.18 **	0.34 ***	0.14 *	0.21 **	0.12	0.05

* = *p* < 0.05 significant; ** = *p* < 0.01 very significant; *** = *p* < 0.001 highly significant.

**Table 5:** Correlations between variables of RIASEC and popularity of teaching topic, *r*(118).

Popularity of Teaching Content	R	I	A	S	E	C
Thinking about oneself	–0.22 **	–0.16 *	0.09	0.16 *	0.10	0.05
Being with others	–0.11	–0.14 *	0.11	0.26 ***	0.07	–0.10
Religions and traditions	–0.15 *	–0.15 *	–0.05	0.04	0.01	–0.02
How products were built	0.10	0.00	0.00	0.07	0.14 *	0.09
Life in earlier generations	–0.20 **	–0.09	0.14 *	0.00	–0.02	–0.08
My hometown	–0.02	–0.13	0.04	0.11	0.09	0.10
Earth, and how people live in other places	0.02	–0.04	0.12	0.03	0.01	0.07
Animals, woods, fields, ponds, and flowers	0.08	0.10	–0.07	–0.11	–0.13	–0.01
Sun, moon, stars, and universe	–0.09	0.00	0.02	–0.03	–0.07	0.00
Water, air, weather, and stones	0.07	0.16 *	–0.03	–0.13 *	–0.07	–0.07
Technology, electricity, and inventions	0.38 ***	0.18 **	–0.25 ***	–0.20 **	–0.12	–0.13
Substances and their properties	0.16 *	0.32 ***	–0.06	–0.18 **	–0.01	0.12

* = *p* < 0.05 significant; ** = *p* < 0.01 very significant; *** = *p* < 0.001 highly significant.

## Data Availability

Not applicabile.
